# Phycobacteria Biodiversity, Selected Isolation, and Bioactivity Elucidation of New Bacterial Species of Highly Toxic Marine Dinoflagellate *Alexandrium minutum* amtk4

**DOI:** 10.3390/microorganisms13061198

**Published:** 2025-05-24

**Authors:** Xiaoling Zhang, Zekang Pan, Jinkai Zhang, Bingqian Liu, Qiao Yang

**Affiliations:** ABI Group, Phycosphere Microbiology Laboratory, College of Marine Science and Technology, Zhejiang Ocean University, Zhoushan 316022, China

**Keywords:** phycosphere microbiology, algae–bacteria interactions, exopolysaccharides, cultivable phycobacteria, microalgae growth-promoting bacterium, toxic dinoflagellate, *Alexandrium minutum*

## Abstract

Phycosphere niches host rich, unique microbial consortia that harbor complex algae–bacteria interactions with fundamental significance in underpinning most functions of aquatic ecological processes. Therefore, harvesting the uncultured phycobacteria is crucial for understanding the intricate mechanisms governing these dynamic interactions. Here, we characterized and compared microbial community composition of the phycosphere microbiota from six harmful algal bloom-forming marine dinoflagellates, *Alexandrium* spp., and their bacterial associations. Furthermore, based on a combinational enhanced cultivation strategy (CECS) procedure for the selected isolation for cultivable phycobacteria, a new yellow-pigmented bioactive bacterium designated ABI-6-9 was successfully recovered from cultivable phycobacteria of the highly toxic *A. minutum* strain amtk4. An additional phylogenomic analysis fully identified this new isolate as a potential novel species of the genus *Mameliella* within the family *Roseobacteraceae*. The bioactivity evaluation observed that strain ABI-6-9 can significantly promote the cell growth of its algal host and altered the gonyautoxin accumulation profiles in the co-culture circumstance. Additionally, the bacterial production of active bioflocculanting exopolysaccharides (EPSs) by strain ABI-6-9 was also measured after culture optimization. Thus, these findings revealed the potential environmental and biotechnological implications of this new microalgae growth- promoting phycobacterium.

## 1. Introduction

Billion-year-old algae are pivotal primary producers in global aquatic ecosystems and fundamental to life on Earth [1]. Hence, the phycosphere niche, as a microscopic-scale and fluid environment, hosts unique, closely associated microbial inhabitants surrounding the algal cell [2]. They are a unique and widely distributed microbial community, and thus form the algal microbiome within the phytoplankton holobiont called the phycobiont as one core concept of phycosphere microbiology [3]. The phycosphere microbial consortium harbors complex algae–bacteria interactions (ABI), in which nutrient trafficking and signaling molecules are central to these dynamic relationships [4]. Within this metabolic hot spot and interactive interface, exopolysaccharides or extracellular polysaccharides (EPSs) secreted by both interactive sides are regarded as vital component of the algae–bacteria joint extracellular matrix (ECM), which embeds proliferating cells, promotes the formation of a complex and interconnected ECM, facilitates aggregation both during phytoplankton blooms in the nature and in laboratory cultures [5] and colony formation, and physically defines the phycosphere [6]. These interactions usually span mutualism, commensalism, antagonism, parasitism, and competition [7]. The interplaying process often involves cross-kingdom, inter- or intra-species exchanges of nutrients, secondary metabolites, infochemicals, and ever vital gene transfer agents (GTAs). Thus, it underpins most functions of aquatic ecological processes, including primary production, marine snow formation, biogeochemical cycles, and the microbial loop in the oceans [8]. Moreover, it is also a critical player in regulating the biosynthesis of the phycotoxins such as paralytic shellfish poisoning toxins (PSTs) derived from various dinoflagellate species during harmful algal blooms (HABs) [9,10,11]. Regarding those HAB-forming dinoflagellates, the genus *Alexandrium* is a well-known group due to its significant impacts on both environmental and human health because of phycotoxins such as PSTs [12]. Moreover, it is worth noting that, due to chemical complexity and bioactivity with remarkable selectivity and ligand affinity toward varied pharmacological targets, these natural neurotoxins provide a successful and promising future for new drug development, such as analgesic pharmaceuticals [7,8,13,14,15].

Microorganisms with a huge biomass and species are an indispensable part of mankind’s production and the life of human civilization [16]. Although pure microbial culture was invented nearly one and a half centuries ago, the phenomenon of “the great plate count anomaly” remains the main obstacle for successfully transferring rich microbial resources to fully serve human society’s development [17]. Currently, there are still only a minority of microorganisms that can be cultured under laboratory conditions, thus leaving the vast bioprospecting potential of the uncultured microorganisms, which are called microbial dark matter (MDM), largely unexplored [18,19,20]. Modern multiomic techniques, such as genomics, transcriptomics, proteomics and metabolomics, etc., have gained increasingly rapid development; however, they still cannot fully help to answer those basic and pivotal questions about the microbes, such as what nutrients the bacteria need, what metabolites they produce, and how they interact with other living organisms in nature [21,22,23,24,25]. In order to find satisfactory answers to these questions, microbiologists are still dedicated to first isolate and culture these microorganisms in the laboratory [26]. So, continuous progress was achieved for microbial culturability enhancement, and new cultivation technologies were successively invented and applied, including co-culture [27,28,29], high-throughput culture [30], trap in situ culture screening [31], and soil matrix membrane system [32]. The invention and application of these innovative technologies significantly increased microbial culturability, resulted in the discovery of huge number of new microbial species, and consequently improved microbial species diversity and promoted greater microbial resource development and wide practical applications during the development of phycosphere microbiology [33].

For the phycobacteria community, it is crucial to expand the ability to cultivate phycobacteria to enhance the understanding of the underlying interactive mechanisms between algae and phycobacteria and promote their multiple applications for human welfare [34]. Previously, we have performed the Phycosphere Microbiome Project (PMP) to characterize the microbial consortium composition of diverse HAB-forming marine dinoflagellates [35,36,37,38,39,40,41,42,43]. In this study, we presented the comparative study of the characterizations of the phycosphere microbiota (PM) of six marine dinoflagellate *Alexandrium* spp. and identified potential bacterial associations. Furthermore, we isolated and conducted a phylogenetic study of a new bacterium designated ABI-6-9 using the combinational enhanced cultivation strategy (CECS) for the selected isolation of cultivable phycobacteria [20,21,22,23,24,25,26]. Additionally, various bioactivities of strain ABI-6-9 were measured, and revealed its multiple roles as a new microalgae growth-promoting bacterium (MGPB) [12,35] and a producer of bacterial bioflocculanting EPSs derived from the phycosphere niche.

## 2. Materials and Methods

### 2.1. Algal Strains and Culture

Six strains of the marine dinoflagellate *Alexandrium* spp. were used in this study. These algal strains were all sampled during the occurrence of natural HABs [44]. *A. tamarense* strain AT260 was obtained from the Yangtze Estuary in the East China Sea [45], *A. minutum* amtk4 and AMKS5 were isolated from southern Taiwan [46,47,48]; *A. catenella* ACHQ and ACHK were originally isolated from the Qingdao sea area in the East China Sea and Hongkong in South China Sea, respectively. *A. tamarense* strain AT11 was isolated from Ireland, UK. The dinoflagellates used in this study were cultured at 20–25 °C in F/2 media with 12L/12D cycles under a light intensity of approximately 25 μmol photons m^−2^ s^−1^ [49,50]. Obtaining the axenic culture of *A. minutum* amtk4 for the algae–bacteria co-culture experiment was performed by repeated washing, lysozyme, SDS, and multiple antibiotic treatments, as described previously [51,52,53].

### 2.2. 16S rRNA Pyrosequencing Analysis

Total genomic DNA was extracted using Fast Bacterial Genomic DNA Extraction Kits (Merck, Shanghai, China) according to manufacturer’s instructions, and the extracted DNA concentration and purity were evaluated by a NanoDrop UV-Vis spectrophotometer (BioTek, Winooski, VT, USA) [54]. A general bacterial primer pair 338F/806R was used to amplify the V3-V4 variable region of bacterial 16S rRNA genes [54]. PCR products were purified by 2% (*w*/*v*) agarose gel electrophoresis. The library was constructed using a TruSeq^®^ DNA PCR-Free Sample Preparation Kit (Illumina, San Diego, CA, USA) purchased from Illumina, and then sequenced using NovaSeq 6000 PE250 platform [55]. Data analysis was performed using qiime2 document (https://docs.qiime2.org, accessed on 3 March 2025). The parameters used were all the default settings. Based on the relative abundance of the main microbial species in the samples, the co-occurrence network analysis was used to calculate the Spearman rank correlation coefficient. The network diagram was drawn using the “igraph” package [56].

### 2.3. Isolation of Cultivable Phycobacteria Using the CECS Procedure

For cultivable phycobacterial isolation, different isolation media were prepared for the combinational enhanced cultivation strategy (CECS), as previously described [57]. Briefly, all isolation media contained 5.24 g/L HEPES (Merck, Shanghai, China) as a buffering agent and 300 mL/L concentrated artificial seawater and were adjusted to pH 7.2 ± 0.1. After sterilization by autoclaving, the media were supplemented with 5.0 mL/L respective algal culture filter [58]. The nanoscale designed composite gel (DCG) was pre-treated with varied micro-nutrients according to the procedure previously described [59]. Each of the triplicate of 1 mL micro-wells were seeded with 10 μL of diluted bacterial cultures which were serially diluted of 1:10 by the dilution-to-extinction technique [60]. These microplates were then incubated at 28 °C for at least 3–4 weeks until visible bacterial colonies growing on the isolation plates were observed. The isolated strains were further purified, maintained on marine agar (MA; Difco, BD, Franklin Lakes, NJ, USA), and preserved at 4 °C as a solid slant for short-term preservation or as a glycerol suspension (25%, *v*/*v*) at −80 °C for long-term preservation.

### 2.4. Phylogenetic Analysis of the 16S rRNA Gene of Strain ABI-6-9

The phylogenetic identification and calculation of pairwise similarities of 16S rRNA gene sequences of the new bacterial strains were achieved using the EzTaxon-e server (http://eztaxon-e.ezbiocloud.net, accessed on 2 March 2025). The sequence alignments were performed using CLUSTAL_X (http://www.clustal.org, accessed on 1 March 2025). Phylogenetic trees were reconstructed with the neighbor-joining (NJ) algorithm using MEGA software (version 7.0) (https://www.megasoftware.net, accessed on 6 March 2025).

### 2.5. Phylogenomic Analysis by ANI, AAI, and dDDH Calculations

Phylogenomic calculations based on average nucleotide identity (ANI) analyses and average amino acid identity (AAI) were performed using the ANI/AAIMatrix Genome-based distance matrix calculator (http://enve-omics.ce.gatech.edu/g-matrix, accessed on 12 March 2025). The digital DNA-DNA hybridization (dDDH) analysis was performed using the Genome-to-Genome Distance Calculator (GGDC 2.1) (http://ggdc.dsmz.de, accessed on 10 March 2025). The phylogenomic tree was constructed using the UBCG pipeline [61].

### 2.6. Bacterial Growth Measurement

For bacterial culture optimization, 1 mL of a 24 h fresh bacterial culture of strain ABI-6-9 was taken and mixed with 24 mL of fresh 2216 medium. Then, 150 μL of the mixture was added into the wells of the 96-well microplate containing different carbon sources, and then cultured with shaking at 60 rpm/min [62]. During different culture periods, the change in bacterial growth was measured at OD_600nm_ every four hours. Ten carbon sources, including cellobiose, fructose, galactose, glucose, glycerol, lactose, maltose, mannose, sucrose, and trehalose, and various pH values (5.0–9.0) were used for the measurements [58,62].

### 2.7. Characterization of the Monosaccharides of Bacterial EPSs

The extraction of bacterial EPSs and chemical elucidation of monosaccharides of crude polysaccharides were performed as previously described using an Agilent 1100 HPLC system (Agilent Technologies, Santa Clara, CA, USA) [63]. Analytical standards (HPLC purity ≧ 99%) of six monosaccharides were purchased from Merck (Shanghai, China).

### 2.8. Evaluation of Bioflocculation and MGP Bioactivity

Bioflocculating activity measurements of bacterial EPSs were performed according to the procedures reported previously [64]. The prepared EPSs were dissolved in distilled water for further measurements. The kaolin clay suspension flocculation (KCSF) assay calculated the bioflocculation rate (BR, %) and was used for the bioactivity measurement using 96-well microplates with at least six replicates per sample [65]. For the MGP activity assay, the preparation of axenic amtk4 cells for co-culture was performed as described previously [66]. Bacterial growth was recorded by monitoring OD_600nm_ changes using a SpectraMax M2 model 96-well microplate reader (Molecular Devices, LLC, San Jose, CA, USA). Measuring the changes in cell numbers of algal strain amtk4 was performed using a Millicell^®^ Disposable hemocytometer (Merck KGaA, Darmstadt, Germany).

### 2.9. Statistical Analysis

All the results are expressed as the means ± SDs. The correlation coefficients (*R*^2^) of the monosaccharide portions with bioflocculanting activities and the statistical significance were performed using Spearman’s rank order correlation analysis and plotted with the OriginPro (Version 10.0) (OriginLab Corp., Northampton, MA, USA). All the obtained results were expressed as means ± SDs. A *p*-value of less than 0.05 was considered to be statistically significant for all analyses.

## 3. Results and Discussion

### 3.1. Characterization of the PM Compositions of Six Alexandrium *spp.*

Based on the pyrosequencing analysis, the PM compositions of six *Alexandrium* spp. were compared. It could inferred that proteobacteria was the predominant group at the phylum level, with a relative abundance range of 32.2–97.2%. However, for *A. tamarense* AT.11, firmicutes was the most prevalent and constituted about 53.6% of the total PMs (Figure S1A). At family level, the *Rhodobacteraceae* was the most prevalent and occupied 10.1–62.1% of total PMs (Figure S1B). With the exception of toxic *A. catenella* ACHK, the family *Flammeovirgaceae* dominated the total PMs (25.5%), followed by *Rhodobacteraceae* (6.5%). Furthermore, among the top ten genera, *Mameliella*, *Sulfitobacter*, *Dinoroseobacter*, and *Marivita* showed significant differences between the toxic and non-toxic groups among the six *Alexandrium* spp. Regarding the second-dominant group of cyanobacteria, only one genus, *Microcystis* was found. It may suggest that cryptic cyanobacterial lineages may be concealed within the dinoflagellate host [54]. Additionally, it is noteworthy that about 16–64% of OTUs were classified as unidentified taxonomic groups (Figure S1B). It clearly indicates that the six PMs of *Alexandrium* spp. harbored great potential for discovering novel species with promising ecological and biological functions.

For the PM of highly toxic *A. minutum* amtk4, proteobacteria (80.9%) was identified as the dominant fraction, followed by bacteroidetes (17.9%) (Figure S1A). At family level, *Alteromonadaceae* occupied about 24.9% of the total PM, followed by *rhizobiaceae* (21.1%), *rhodobacteraceae* (16.4%), *cyclobacteriaceae (*16.3%), unidentified *cohaesibacter* (2.6%), and *sphingomonadaceae (*1.5%) (Figure S1B). Moreover, four dominant genera were identified: *Mameliella* (30.1%), *Alteromonas (*24.9%), *Cohaesibacter (*20.5%), and *Ekhidna (*15.8%) (Figure S1C). In particular, *Mameliella* exhibited a higher abundance. In addition, the highest portion of 64% of OTUs were classified as unidentified groups in the six PMs of *Alexandrium* spp. This considerable observation highlights the need for further investigation to fully understand complete diversity and potential roles of these PMs in the phycosphere ecosystem of HAB-causing marine *Alexandrium* spp.

### 3.2. Analysis of Bacterial Associations in the PMs of Six Alexandrium *spp.*

The co-occurrence of microorganisms can be modeled using a network analysis to illustrate complex microbial relationships and responses to variations in operational factors and suggest the clustering of sub-communities, and more detailed information also can be provided to reveal microbial interactions and their functional correlations with ecosystems [67]. Based on the co-occurrence network analysis, it is noteworthy that *Mameliella*, which was one abundant genera of the six PMs, showed significant positive correlations with *Roseovarius*, *Thalassobacter*, *Phaeobacter*, and *Muricauda*. Moreover, three dominant genera—*Mameliella*, *Roseovarius*, and *Muricauda*—demonstrated close positive associations with each other. Interestingly, it is also found that *Mameliella* demonstrated a relatively low abundance (<1.0%) in the non-toxic group (AT-11, AMKS5, and ACHQ). Otherwise, it was processed as absolutely abundant portions in the toxic group (AMTK4, AT 260, and ACHK). Considering the potential function of the *Mameliella* species during algae–bacteria interactions [68], the reason governing for this specific distribution pattern of the *Mameliella* is well worth further investigation.

### 3.3. CECS-Based Selected Isolation of Cultivable Phycobacterial Strains

The realization of pure cultures of phycobacteria provides a vital basis and necessary prerequisite for the successful construction of co-culture system and further explaining the detailed mechanisms governing algae–bacteria interactions [9,10,11]. To achieve this purpose, a selected isolation procedure named CECS was proposed in our previous study [25]. Based on CECS isolation, total 16 phycobacterial strains were recovered from the *A. minutum* amtk4 culture (Figure S3A). To determine the phylogenetic relationship, a phylogenetic tree using the 16S rRNA gene was constructed (Figure S3A). Strain ABI-6-9 was clustered with strain LZ-21, which was isolated from the toxic dinoflagellate *A. catenella* LZT-09 [25]. Among these new isolates, eight bacterial strains have been identified as novel species [13,35,40,41,42,43,44] (Figure S3B). This findings indicated that the proposed CECS procedure is indeed effective for harvesting the uncultured phycobacteria. Thereafter, it will lay solid basis for furthering studies on algae–bacteria interactions to fully understand their intricate mechanisms.

### 3.4. Phylogenetic Analysis of Strain ABI-6-9

**Figure 1:**
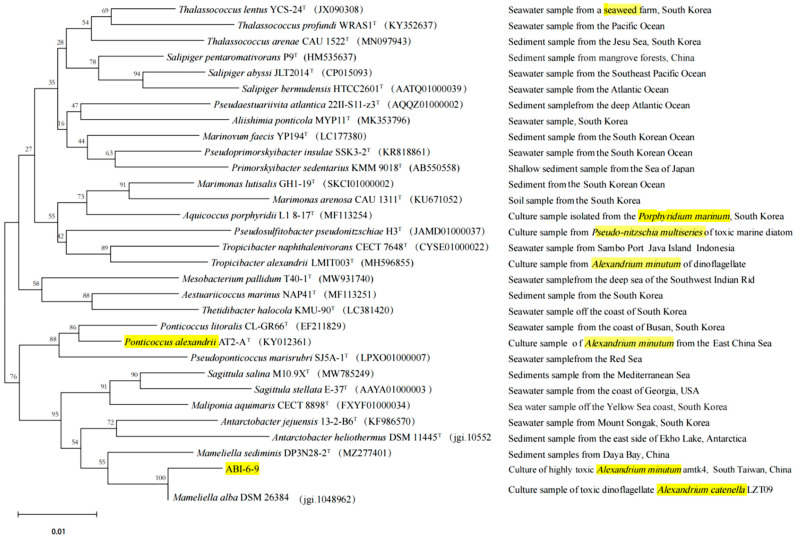
Phylogenetic tree constructed based on the bacterial 16S rRNA gene of strain ABI-6-9 and the closely related type strains of the families *Rhodobacteraceae* and *Roseobacteraceae*, and the isolation sources. Those phycobacterial strains were indicated in yellow color.

The phylogenetic analysis based on the 16S rRNA gene revealed that strain ABI-6-9 exhibited the gene similarity of 97.98% with *Mameliella alba* JLT354-W^T^ [68]. The value was less than the threshold value (98.65%) for novel species identification [58]. The phylogenetic tree was then constructed. It can be seen that strain ABI-6-9 formed a distinct separate phylogenetic line and clustered with *M. alba* JLT354-W^T^ (Figure 1). Based on these data, it can be inferred that strain ABI-6-9 represents a potentially new species within the genus *Mameliella* of family *Roseobacteraceae*. Additionally, it is interesting to note that among the closely related type species of strain ABI-6-9, four bacteria, including *Thalassococcus lentus* YCS-24^T^, *Pseudosulfitobacter pseudonitzschiae* H3^T^, *Tropicibacter alexandrii* LMIT003^T^, and *Ponticoccus alexandrii* AT2-A^T^, were also isolated from phycosphere niches.

### 3.5. Phylogenomic Characterization of Strain ABI-6-9

To further infer the phylogenetic relationship of strain ABI-6-9, a phylogenomic tree using an up-to-date bacterial core gene set (UBCG) was performed. As shown in Figure S4, strain ABI-6-9 was phylogenetically attributed to the genus *Mameliella* and clustered with the type species of *M. alba* JLT354-W^T^. Additionally, three main phylogenomic parameters, including ANI, AAI, and dDDH values, were calculated as 93.7%, 92.1%, and 65.5%, respectively. All the values were below the thresholds (95–96% for ANI, 97% for AAI, and 70% for dDDH) generally accepted for new species delineations [34]. Accordingly, it confirmed that strain ABI-6-9 was a new member and a potential novel species of the genus *Mameliella*.

### 3.6. Microalgae Growth-Promoting Potential Analysis of Strain ABI-6-9

**Figure 2:**
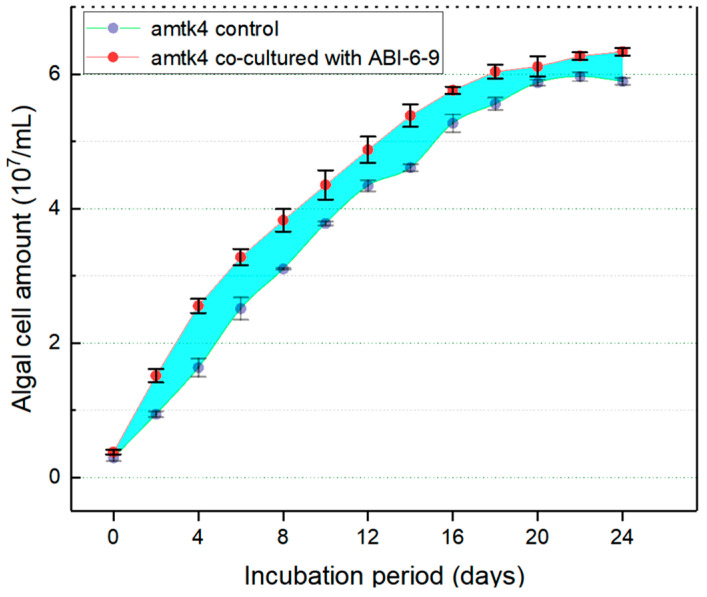
Evaluation of the MGP potential of strain ABI-6-9 on algal host *A. minutum* amtk4 cells.

Recently, MGPBs have received considerable attention due to their potential to develop microalgae–bacteria co-culture strategies for improved efficiency and sustainability of the water–energy–environment nexus [69,70]. Based on MGP bioactivity assays, the cell numbers of amtk4 increased significantly when directly co-cultured with strain ABI-6-9, and demonstrated an MGP efficiency of at least 16.5 ± 3.8% (Figure 2). This observation clearly suggests potential close associations between strain ABI-6-9 and its algal host, while the precise nature of the mechanism remains unresolved. Previous studies have revealed that *Mameliella alba* can alleviate vitamin limitations of its algal host *Karenia brevis*, a toxigenic marine HAB-forming dinoflagellate, by providing vitamins B_1_, B_7_, and B_12_ and thus greatly contributes to algal growth dynamics [30,48]. Current ongoing research exploring associations under algae–bacteria co-culture conditions using multiomics analysis is expected to provide new insights into the mechanisms governing the interactions between strain ABI-6-9 and its algal host.

### 3.7. Promotion of the Accumulation of Algal GTXs by Algal–Bacterial Co-Culture

**Figure 3:**
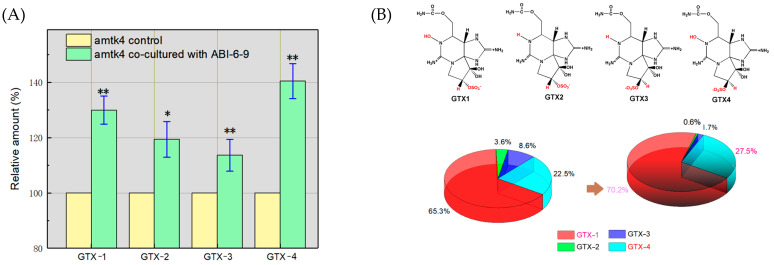
Enhanced effect of algal–bacterial co-culture on the accumulation (**A**) and changes in the individual portions (**B**) of four algal GTX toxins, which are indicated with their chemical structures. Notes: *, *p* < 0.05 and **, *p* < 0.01.

The HPLC analysis of algal GTX toxin production indicated that the total toxin production by algal amtk4 was about (1.83 ± 0.05) × 10^−14^ mol/cell, and that of the algal–bacterial co-culture was determined as about (2.48 ± 0.34) × 10^−14^ mol/cell (Figure 3A). It showed that the co-culture manner obviously promoted algal GTX accumulation up to about 1.36 times compared with that of algae alone. Furthermore, it is observed that algal–bacterial co-culture also changed the individual portions of algal GTXs (Figure 3B). Among the four algal GTX toxins produced, the contents of GTX-1 and GTX-4 were much higher than those of GTX-3 and GTX-2 under both culture conditions. However, in algal–bacterial co-culture, the contents of the GTX-1 and GTX-4 toxins (more toxic) increased from 87.8% to 97.7% (about 1.13 fold, *p* < 0.01) among the total toxins, Accordingly, those of the GTX-2 and GTX-3 toxins (less toxic) were accordingly decreased from 12.2% to 2.3% in the total (*p* < 0.01). These findings may provide a useful clue to help optimize and enhance the biological production of those promising phycotoxins [65,66]. It also may help us to understand the implications for the environmental toxicity of phycotoxins such as PSTs [6,7,8,9,10].

### 3.8. Culture Optimization of EPS Production by Strain ABI-6-9

**Figure 4:**
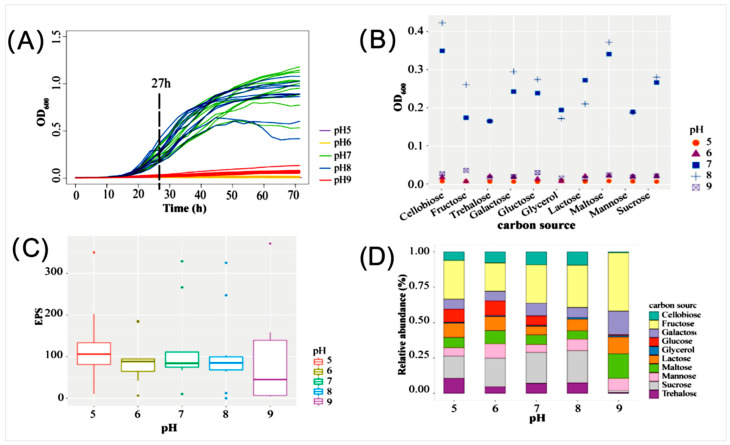
Culture optimization of bacterial growth and EPS production of strain ABI-6-9. Effects of different pHs and carbon sources on bacterial growth recorded at OD_600nm_ (**A**,**B**) and EPS production (**C**,**D**).

EPSs are regarded as vital component of the algae–bacteria joint extracellular matrix [6,7,8,9]. A preliminary investigation indicated that the pH and carbon source in the media were the primary factors influencing EPS accumulation [24,70]. In this study, ten selected carbon sources, including cellobiose, fructose, galactose, glucose, glycerol, lactose, maltose, mannose, sucrose, and trehalose, and a pH range (5.0–9.0) were used for culture optimization. The obtained result showed that strain ABI-6-9 exhibited better growth when cultured at a pH ranging from 7.0 to 8.0 (Figure 4A). Additionally, the fastest bacterial growth of strain ABI-6-9 was achieved when cellobiose was used as the sole carbon source in culture medium and cultured at pH 8.0 (Figure 4B). However, the EPS accumulation indicated that the highest EPS yield was obtained when cultured at pH 9.0 (Figure 4C). Consequently, under the optimized conditions, the highest EPS yield of 115.6 ± 17.8 μg/mL was achieved when cellobiose (10 g/L) was used as the carbon source and bacteria were cultured at 28 °C, pH 9.0 (Figure 4D). It indicated that the increasing the medium pH may enhance bacterial EPS accumulation in strain ABI-6-9. It is speculated that in response to shifts in extracellular pH, bacteria usually secrete extra EPSs to cover the cellular surface, thereby mitigating adverse effects on cellular survival and function [10,11,12].

### 3.9. Bioflocculanting Bioactivity of EPSs Produced by Strain ABI-6-9

Polysaccharides play a fundamental roles in the phycosphere niche either by providing necessary valuable carbon sources for the phycobacteria community [5,6] or ensuring the formation of a favorable microenvironment for attachment, maintaining exoenzyme activity, sequestering nutrients, and protecting against toxins [7]. Additionally, they demonstrated promising bioflocculanting activity [6,7,8,9]. Phycobacterial candidates acting as natural EPS producers also possess varied overwhelming advantages, such as fast bacterial growth, flexible environmental adaptability, and convenient engineering operation for genetic modification with an easily accessible and clearer genetic background for biological and industrial applications [6].

**Figure 5:**
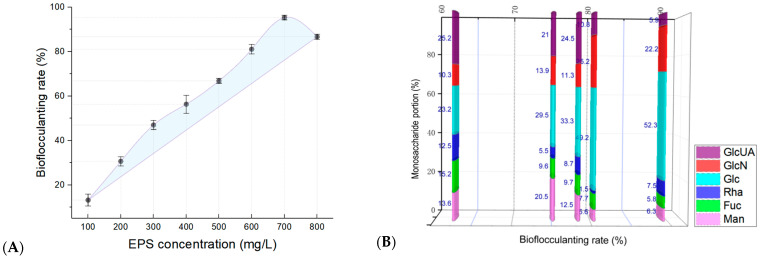

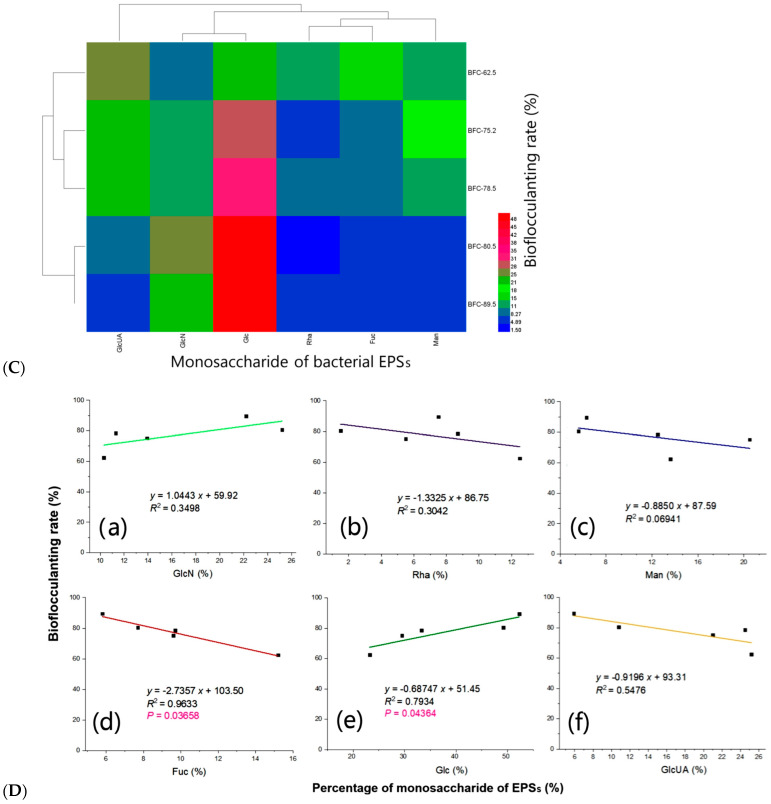
Bioflocculanting activity profiles of bacterial EPSs produced by strain ABI-6-9 (**A**), the monosaccharide portion of bacterial EPSs (**B**), their overall associations with bioflocculanting activity visualized in a clustered heat map (**C**), and the correlation analysis of the relative portions (%) of six monosaccharides (**D**), including *D*-galactosamine (GlcN, panel **a**), *L*-rhamnose (Rha, panel **b**), mannose (Man, panel **c**), *D*-fucose (Fuc, panel **d**), glucose (Glc, panel **e**), and *D*-galacturonic acid (GlcUA, panel **f**), with the bioflocculanting rate (%).

In this study, to evaluate the production capacity of bacterial bioflocculants, the EPSs produced by strain ABI-6-9 were extracted and then subjected to a bioflocculanting activity evaluation. A comparison of the bioflocculanting effect under a series of EPS concentrations was performed. As shown in Figure 5A, the bioflocculanting efficiency of bacterial EPS showed a concentration-dependent manner, and it reached a maximum of 95.6 ± 7.5% (mean ± SD) when the EPS concentration was 0.70 g/L [39,40]. Previously, we found that the composition of EPSs varied considerably between different phycobacterial strains, and potentially reflected their fate in the extracellular environment during algae–bacteria interactions [12,22,23,32]. To further infer whether the type and composition of monosaccharides of EPSs were related to the bioflocculanting activity, characterizations of the monosaccharide composition (Figure 5B) and the correlations (Figure 5C) with bioflocculanting activity were performed. Based on the constructed heatmap, the relative portions of glucose demonstrated a significantly positive correlation with the bioflocculanting capacities, with correlation coefficient of (*R*^2^) of 0.7934 (*p* = 0.04364), while the portion of fucose showed an obvious negative correlation with a high *R*^2^ value of 0.9633 (*p* = 0.03658). In addition, the other three monosaccharides (Rha, Man, and GlcUA) were found to be negatively correlated, and GlcN had a positive correlation, but all were without statistical significance (Figure 5D). Despite these findings, the detailed chemical structures of bacterial EPSs remain to be further elucidated. Thus, these finding indicated that the new isolate, strain ABI-6-9, may serve as a new bacterial candidate with natural potential for the production of promising microbial bioflocculants derived from the phycosphere niche [35,36,37,38,39,40,41,42,71,72,73,74].

## 4. Conclusions

Characterizing the microbial composition of the phycosphere microbiota of six marine HAB-forming dinoflagellate *Alexandrium* spp. revealed they harbored vast portions of unidentified taxonomic groups and may offer attractive potential for discovering novel species with promising ecological and biological functions. Some cryptic cyanobacterial lineages within the dinoflagellate host were also found within the phycosphere community. To expand the number of new cultivable phycobacteria, a yellow-pigmented bacterium designated ABI-6-9 was recovered from highly toxic *A. minutum* amtk4 based on the CEFC procedure for the selected isolation of the phycobacteria. The phylogenomic analysis confirmed the phylogenetic position of this new isolate to represent a potential novel species of the genus *Mameliella* within the family *Roseobacteraceae*. Strain ABI-6-9 demonstrated an obvious promoting potential toward algal cell growth, both of the gonyautoxins’ accumulation profiles and their individual portions. The bacterial accumulation of active bioflocculanting EPSs indicated that the portions of glucose had a positive correlation with the bioflocculanting capacity, and fructose had a negative association. This study highlights the environmental and biotechnological implications of a newly discovered microalgae growth-promoting bacterium recovered from the phycosphere niche.

## Data Availability

The original contributions presented in this study are included in the article. Further inquiries can be directed to the corresponding author.

## Supplementary Materials

The following supporting information can be downloaded at https://www.mdpi.com/article/10.3390/microorganisms13061198/s1: Figure S1. Distribution patterns of bacterial abundance at the phylum and family levels of the phycosphere microbiota of six *Alexandrium* spp. (A) and the relative bacterial abundance distributions of the top 10 genera of the family *Rhodobacteraceae* (B). Samples: the non-toxic group, *A. tamarense* AT-11, *A. minutum* AMKS5, and *A. catenella* ACHQ, and the toxic group: *A. minutum* AMTK4, *A. tamarense* AT 260, and *A. catenella* ACHK. Figure S2. Constructed co-occurrence networks at the genus level of the PM of highly toxic *A. minutum* amtk4. Circle size reflects the number of connections of one specific OTUs with the others. Green lines were for positive associations, and the red lines were for the negative. The genera of *Mameliella* was indicated in red. Figure S3. The constructed phylogenetic tree based on bacterial 16S rRNA gene sequences of cultivable phycobacterial strains isolated from *Alexandrium* spp. (A), and type species established by our laboratory (B). Bacterial strains isolated from highly-toxic *A. catenella* amtk4 were indicated in yellow color. Figure S4. Constructed phylogenomic tree based on the up-to-date bacterial core gene (UBCG) set between strain ABI-6-9 and closely related type strains within the families *Rhodobacteraceae* and *Roseobacteraceae*. Five typical genome parameters including genome size, GCC%, and the numbers of rRNA, tRNA and coding DNA sequence (CDS) were also shown in the right. *Bar*: 0.1 nt substitutions per site.

## Abbreviations

The following abbreviations are used in this manuscript:

AAI	average amino acid Identity
ABI	algae-bacteria interactions
ANI	average nucleotide identity
CECS	combinational enhanced cultivation strategy
dDDH	digital DNA-DNA hybridization
DCG	designed composite gel
ECM	extracellular matrix
EPS	exopolysaccharide
GTAs	gene transfer agents
GTX	gonyautoxin
HABs	harmful algal blooms
HEPES	4-(2-hydroxyethyl)-1-piperazineethanesulfonic acid
IAA	indole-3-acetic acid
MA	marine agar
MDM	microbial dark matter
MGP	microalgae growth-promoting
MGPB	microalgae growth-promoting bacterium
OD	optical density
OTUs	operational taxonomic units
PM	phycosphere microbiota
PMP	Phycosphere Microbiome Project
PSP	paralytic shellfish poison
PSTs	paralytic shellfish poisoning toxins
UBCG	up-to-date bacterial core gene set
