# Supplementary material for: Phycobacteria Biodiversity, Selected Isolation, and Bioactivity Elucidation of New Bacterial Species of Highly Toxic Marine Dinoflagellate Alexandrium minutum amtk4
Source: Microorganisms. 2025 May 24;13(6):1198. doi: 10.3390/microorganisms13061198 (PMC12195228; doi:10.3390/microorganisms13061198)

Supplementary Materials

Phycobacteria Biodiversity, Selected Isolation, and Bioactivity Elucidation of New Bacterial Species of Highly Toxic Marine Dinoflagellate *Alexandrium minutum* amtk4

Xiaoling Zhang, Zekang Pan, Jinkai Zhang, Bingqian Liu and Qiao Yang *

ABI Group, Phycosphere Microbiology Laboratory, College of Marine Science and Technology, Zhejiang Ocean University, Zhoushan 316022, China

* Correspondence: qiaoyang1979@whu.edu.cn

Figure S1. Distribution pattern of bacterial abundance at phylum and family levels of the phycosphere microbiota of six *Alexandrium* spp. (A), the relative bacterial abundances distribution of the top 10 genus of the family *Rhodobacteraceae* (B). Samples: the non-toxic group, *A. tamarense* AT-11, *A. minutum* AMKS5, and *A. catenella* ACHQ, and the toxic group: *A. minutum* AMTK4, *A. tamarense* AT 260, and *A. catenella* ACHK.

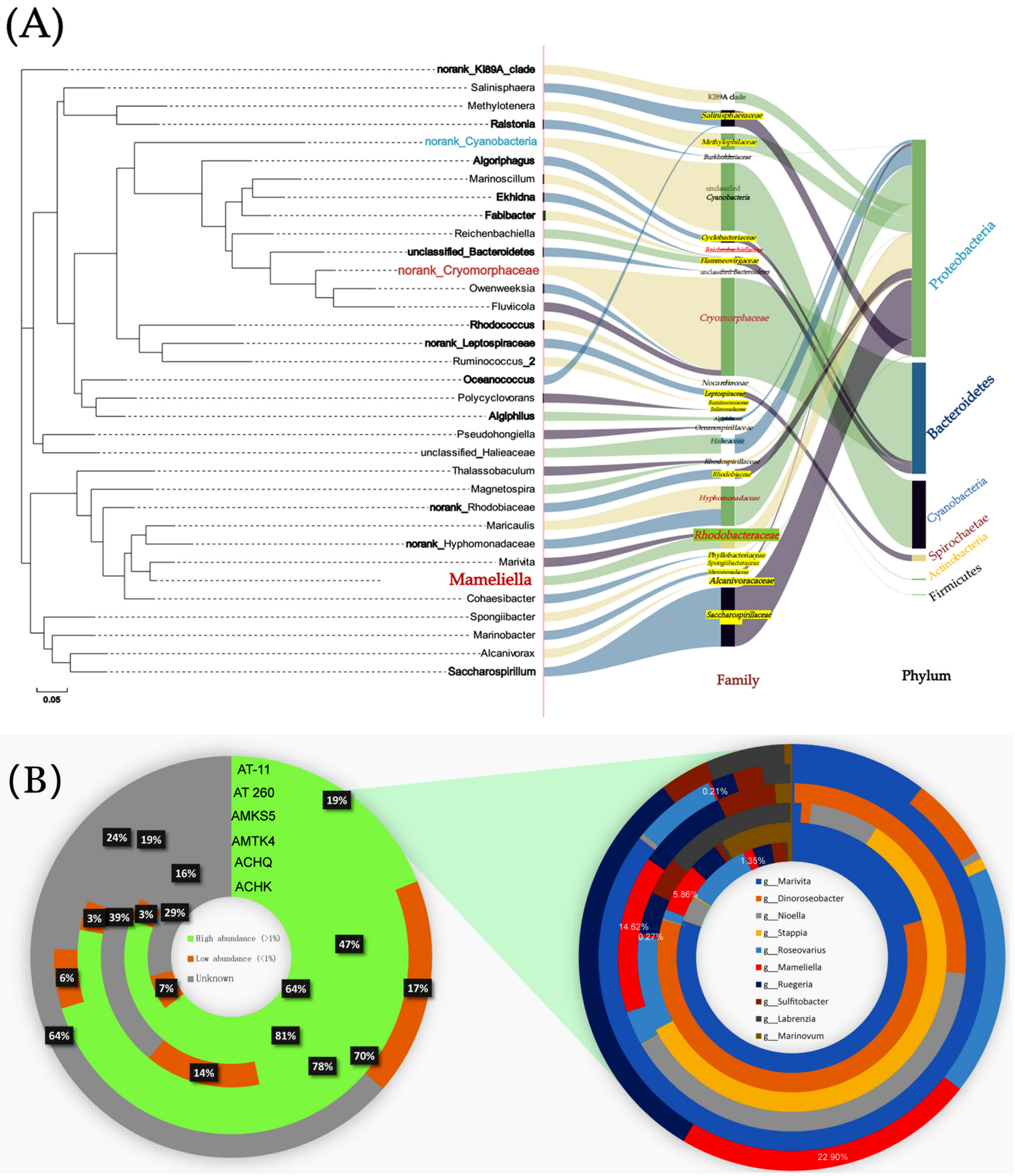

Figure S2. Constructed co-occurrence networks at genera level of the PM of highly-toxic *A. minutum* amtk4. Circle size reflects the number of connections of one specific OTUs with the others. Green lines were for positive associations, and the red lines were for the negative. The genera of *Mameliella* was indicated in red.

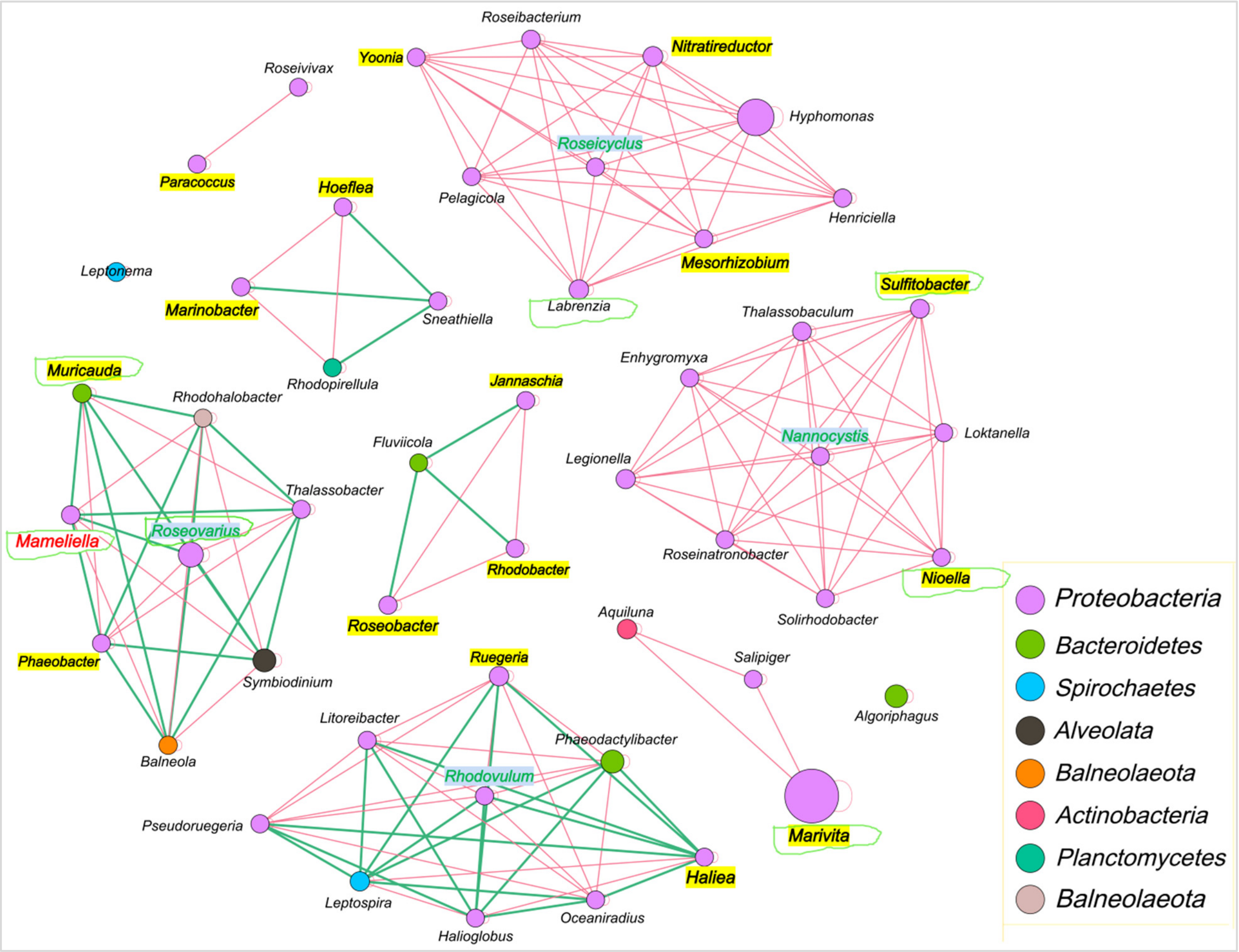

Figure S3. The constructed phylogenetic tree based on bacterial 16S rRNA gene sequences of cultivable phycobacterial strains isolated from *Alexandrium* spp. (A), and type species established by our laboratory (B). Bacterial strains isolated from highly-toxic *A. catenella* amtk4 were indicated in yellow color.

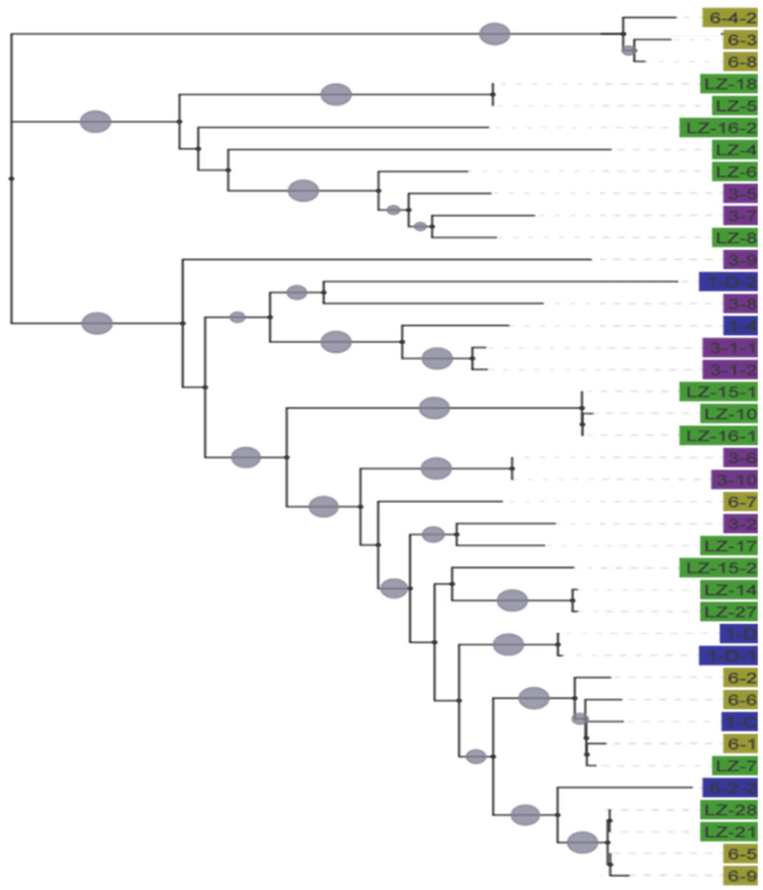

(A)

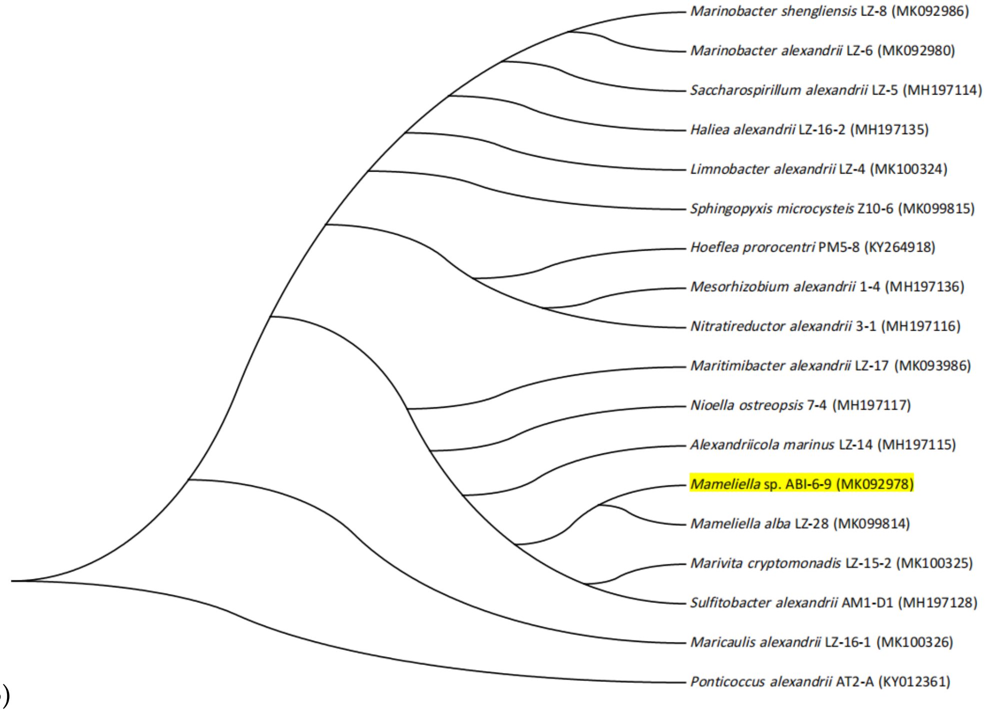

(B)

Figure S4. Constructed phylogenomic tree based on up-to-date bacterial core gene (UBCG) set between strain ABI-6-9 and closely related type strains within family *Rhodobacteraceae* and *Roseobacteraceae*. Five typical genome parameters

including genome size, GCC%, and the numbers of rRNA, tRNA and coding DNA sequence (CDS) were also shown in the right. *Bar*: 0.1 nt substitutions per site.

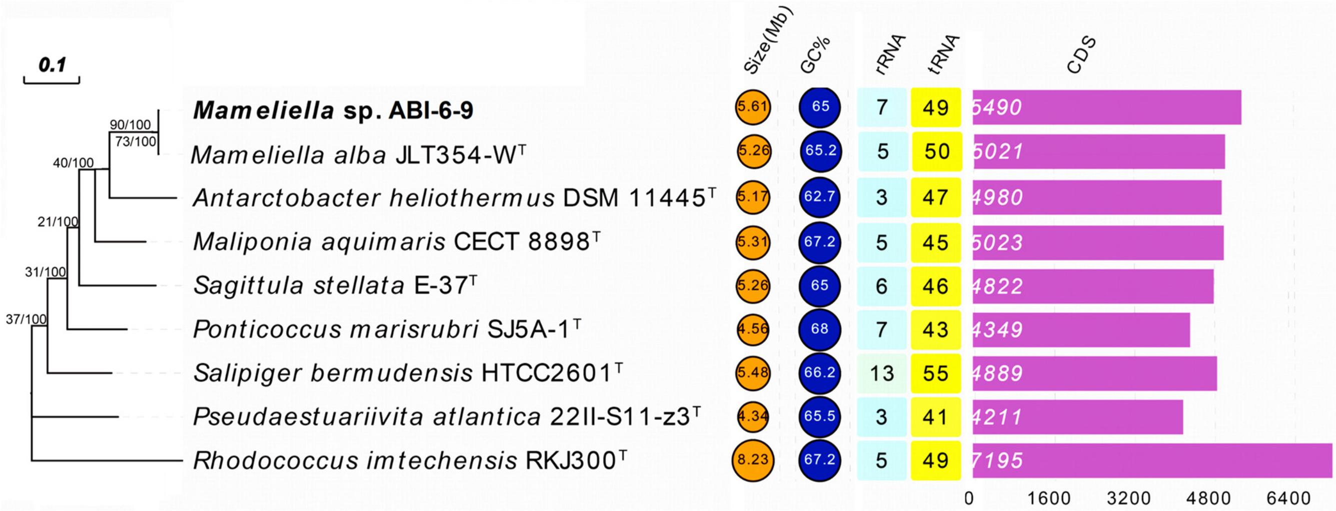

Supplement: Supplementary file 1 [file microorganisms-13-01198-s001.zip › microorganisms-3623571-supplementary.pdf]
